# Electrostatic Clamp and Loop Dynamics Dictate Caspase‑8 Cleavage of the Apoptotic Protein Bid

**DOI:** 10.1021/acs.jpclett.5c01976

**Published:** 2025-07-18

**Authors:** Chien-Lun Hung, Wen-Hsien Wang, Yu-Chuan Chang, Yei-Chen Lai, Yun-Wei Chiang

**Affiliations:** † Department of Chemistry, 34881 National Tsing Hua University, Hsinchu 300-044, Taiwan; ‡ Department of Chemistry, 34916 National Chung Hsing University, Taichung 402-202, Taiwan

## Abstract

The canonical LQTD motif (residues 56–59) has long been regarded as the sole “address tag” that directs Caspase-8 to cleave the pro-apoptotic protein Bid at D59. Here we overturn this view, showing that Bid’s previously uncharted 42-residue “disordered” loop furnishes a second layer of control that can either accelerate or block cleavage. Alanine scanning, MD simulations, DEER spectroscopy, and AlphaFold reveal two physical modules in this loop: an electrostatic clamp formed by E53-D54-E55 that secures the substrate upstream of LQTD, and an entropic flexibility switch at S61-Q62 that modulates catalysis. Phosphorylation at S61 locks the switch and halts proteolysis, whereas a double-alanine substitution (S61A-Q62A) loosens intraloop hydrogen bonding and boosts cleavage by ∼50%. Loop charge, dynamics, and post-translational modification thus cooperate to set Caspase-8 specificity, establishing dynamic loops as tunable physical targets for chemical control of apoptotic signaling.

## Graphical abstract

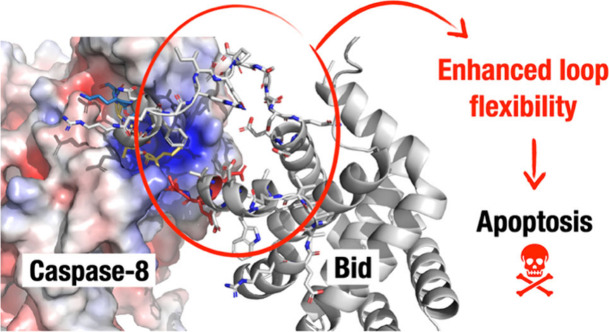

The intrinsic apoptotic pathway counters stresses such as DNA damage, growth-factor withdrawal, heat shock and chemotherapeutic insult by permeabilising the outer mitochondrial membrane (MOMP) and releasing cytochrome *c*.^1,2^ Its execution hinges on the Bcl-2 family, which includes antiapoptotic guardians (e.g., Bcl-2, Bcl-XL) and pro-apoptotic effectors: multidomain Bax/Bak/Bok and BH3-only sensors such as Bid.^2–4^ Bid uniquely bridges extrinsic and intrinsic death signals:^5^ Caspase-8, activated at death receptors, cleaves Bid into p7 and the membrane-active p15 fragment (tBid).^6–9^ The cleaved Bid translocates to mitochondria, binds cofactors such as Mtch2 and cardiolipin, and primes Bax for membrane insertion and oligomerization, culminating in cytochrome *c* release and downstream caspase activation (Figure 1A).^10^

**Figure 1:**
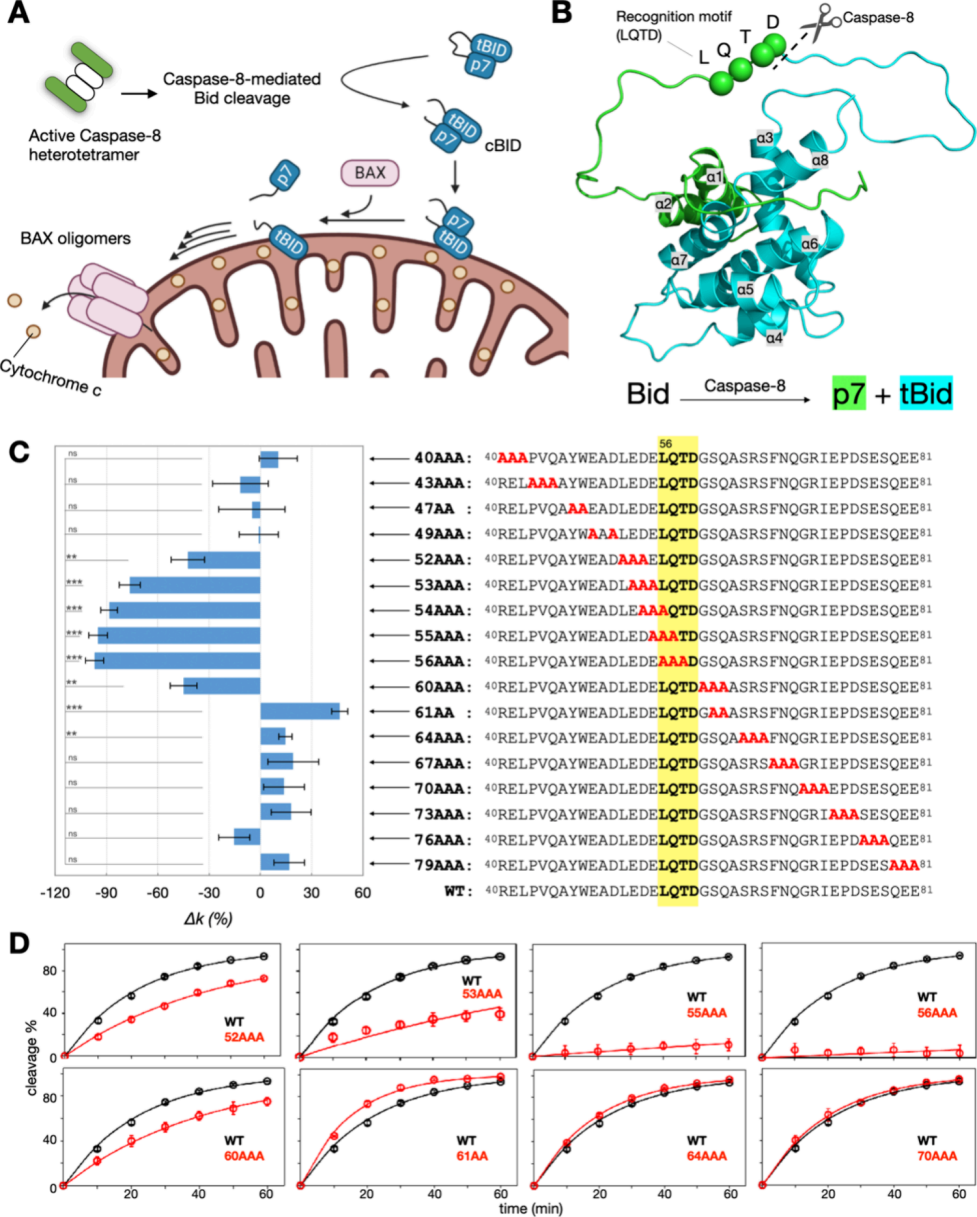
Bid structural models and results of the proteolysis assays. (A) Schematic representation of the roles of Bid and Caspase-8 in apoptotic pathways. (B) Structural model depicting full-length Bid (PDB: 1DDB), highlighting the canonical recognition motif (L56-Q57-T58-D59), the cleavage site (D59), and the resulting fragments postcleavage. (C) Sequence of the long loop in mouse WT Bid along with the alanine mutations studied. The results of the proteolysis assays are presented as relative cleavage rates (Δ*k*), indicating the cleavage rate of each Bid variant relative to WT Bid. *P < 5%; **P < 1%; ***P < 0.1%; ns, not significant. The canonical recognition motif LQTD is highlighted in yellow. (D) Representative experimental data fitting used to evaluate the extent of intact Bid cleaved by Caspase-8 over time. Data are presented as mean ± standard error (*n* ≥ 3 independent experiments). See also Figures S1 and S2 for additional experimental results.

Although Bid is a well-established Caspase-8 substrate, the structural logic that dictates when and how it is cleaved has never been resolved. Both mouse and human Bid share an eight-helix core linked by an unusually long (>40-residue) loop between helices 2 and 3 (Figure 1B).^11,12^ This loop contains the canonical L56-Q57-T58-D59 motif and the Caspase-8 cleavage site Asp59, yet its extraordinary length, dense charge pattern, and putative disorder have left its functional role opaque.^13,14^ Long, apparently “unstructured” loops are common in signaling proteins (including the BH3-only activator PUMA and the antiapoptotic Bcl-2 and Bcl-XL proteins), but whether and how these flexible segments encode specific electrostatic or allosteric signals that direct protease recognition has yet to be systematically dissected.^15–17^

Here we systematically decode the entire loop as a tunable regulatory element. Using >50 alanine-scanning mutants,^18,19^ we mapped residue-level effects on Caspase-8 cleavage kinetics with a specifically developed assay, pinpointing positions that accelerate or inhibit proteolysis. To correlate activity with structure, we employed double electron–electron resonance (DEER) spectroscopy,^20^ an electron-spin-resonance method exquisitely sensitive to nanometre-scale distance changes,^17,20–23^ alongside molecular-dynamics (MD) simulations and AlphaFold structure prediction. This integrative strategy reveals two physical modules in this loop. These insights advance the broader goal of deciphering how dynamic protein segments encode apoptotic signals and lay conceptual groundwork for chemically modulating the Bid–Caspase-8 axis in apoptosis-related diseases.

## Alanine-Scanning Mutagenesis Identifies Critical Residues for Bid Cleavage

To assess the contribution of individual residues within the 42-residue-long loop to the efficiency of Caspase-8-mediated cleavage of Bid, we conducted a comprehensive alanine-scanning mutagenesis using a triple-alanine strategy. We generated various triple-alanine Bid variants (Figure 1C) and subjected them to the SDS-PAGE-based proteolysis assay (see SI Methods) to evaluate the rate of Caspase-8-mediated cleavage. The cleavage rate of each Bid variant was determined by analyzing SDS-PAGE results obtained from different incubation times of Caspase-8 with each Bid variant. These analyses quantified the extent of intact Bid cleaved by Caspase-8 over time (Figure 1D), thus determining the cleavage rate, a key kinetic parameter.

The kinetic analysis results are presented as relative rates (denoted as Δ*k* in Figure 1C), representing the cleavage rate of each Bid variant relative to that of wild-type (WT) Bid, assuming equal amounts of Caspase-8 were used. Positive and negative Δ*k* values indicate that the cleavage rate of Bid by Caspase-8 is greater or slower, respectively, compared to WT Bid. Refer to the Supporting Information (Figure S1) for representative SDS-PAGE results and detailed assay conditions for Caspase-8-mediated Bid cleavage.

Our alanine-scanning results indicate that variants with triple-alanine mutations within the sequence region of 52–60 lead to a significant reduction in the cleavage rate (Figure 1C), consistent with the established role of residues 56–59 as a recognition motif for Caspase-8 in Bid cleavage. Additionally, residues 52–55 appear to be more critical than previously thought, warranting further investigation. Interestingly, the 61AA variant (S61A-Q62A) exhibits a significant increase in the cleavage rate, being 46% higher than that of WT Bid (Figure 1C). Other alanine variants outside this region show insignificant changes in the cleavage rate compared to WT Bid.

## Single-Mutation Study Reveals Roles of Individual Residues

Next, we prepared single-mutation variants focusing on those that caused significant changes in the triple-alanine-scanning study. While the 49AAA variant (E49A-A50A-D51A) caused little change in the cleavage rate, the 52AAA variant (L52A-E53A-D54A) caused a significant reduction (Δ*k* < −42%) in the cleavage rate compared to WT Bid (Figure 1C). This result suggests that residues starting from position 52 are crucial for Caspase-8 recognition and cleavage, a new finding not previously reported in the literature.

To identify the contributions of individual residues near position 52, we prepared single-alanine variants (Figures 2A and S2): L52A, E53A, D54A, and E55A. We found that the L52A mutation improved the cleavage rate (Δ*k* > 15%), whereas other single-alanine mutations (E53A, D54A, and E55A) led to a notable reduction in the cleavage rate (Δ*k* < −24%). This suggests that L52 is relatively less important than residues 53–55 in disrupting the cleavage.

**Figure 2:**
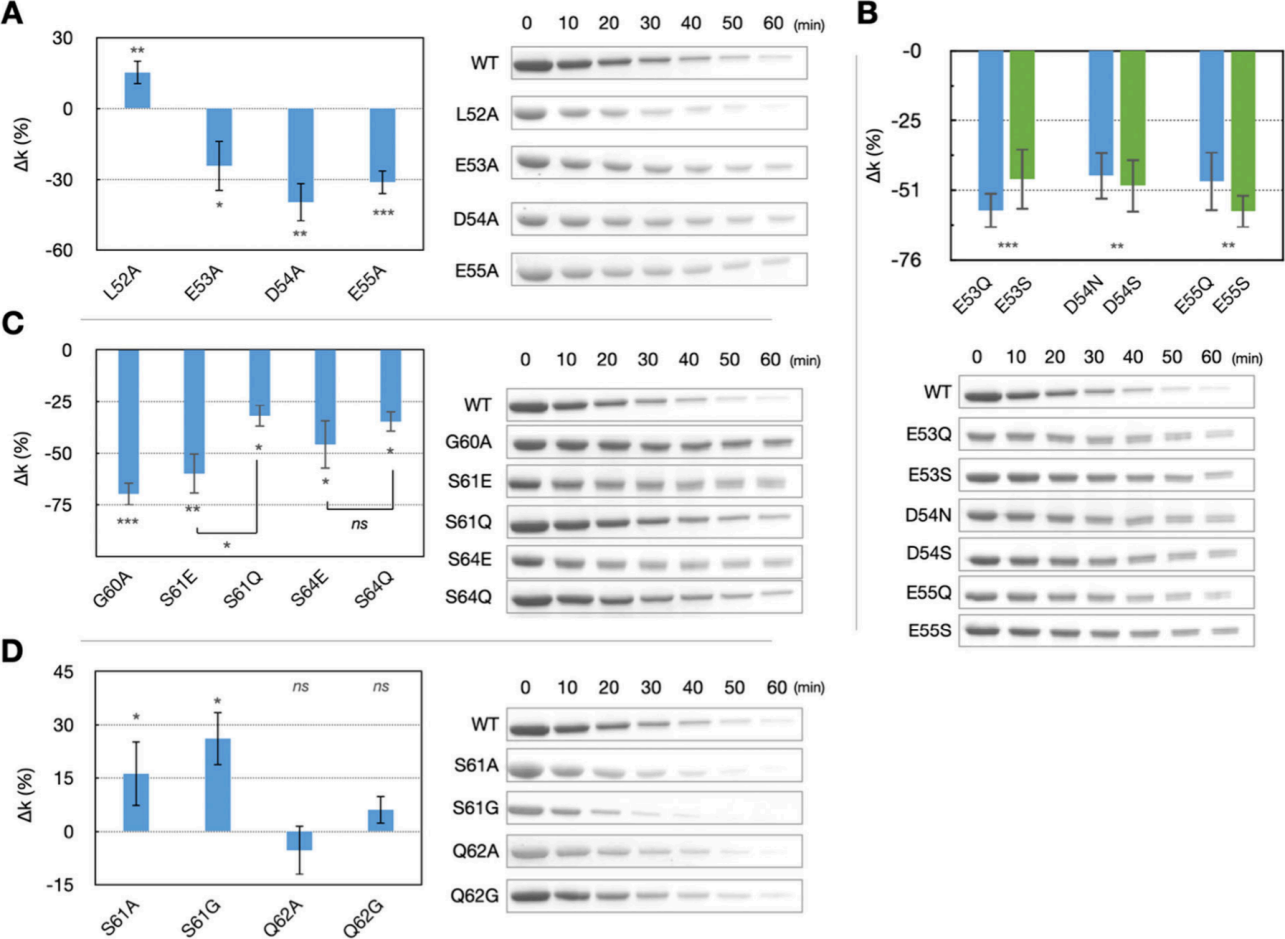
Proteolysis results for single-mutation Bid variants. Shown are the relative cleavage rates (Δ*k*) and representative SDS-PAGE results for single-mutation Bid variants: (A) L52A, E53A, D54A, and E55A. (B) E53Q, E53S, D54N, D54S, E55Q, and E55S. (C) G60A, S61E, S61Q, S64E, and S64Q. (D) S61A, S61G, Q62A, and Q62G. Data are presented as mean ± standard error (*n* ≥ 3 independent experiments). *P < 5%; **P < 1%; ***P < 0.1%; ns, not significant.

To further investigate the importance of the negatively charged residues at positions 53–55 (Figures 2B and S2), we prepared single-mutation variants that either removed negative charges (E53Q, D54N, and E55Q) or reduced side-chain steric hindrance (E53S, D54S, and E55S). The results showed that all these variants led to a significant reduction in the cleavage rate (Δ*k* < −45%). These findings clearly demonstrate that the negatively charged residues E53, D54, and E55 play a crucial role in Caspase-8-mediated cleavage of Bid.

We also studied the G60A variant (Figure 2C). Because residue G60 is adjacent to the cleavage site D59, a small change in side-chain size (i.e., G60A) resulted in a significant reduction in the cleavage rate. Combined with the previously discussed disruptions caused by mutations in the sequences 53–55 and 56–59, our study highlights the critical role of residues 53–60 as a recognition patch for Caspase-8-mediated cleavage of Bid. Consequently, our kinetic data identify residues 53–55 as an essential upstream docking element, establishing 53–60 as the functional recognition patch for Caspase-8. Hereafter, we refer to residues 53–60 as the recognition patch for Bid cleavage by Caspase-8.

## Single-Mutation Study Downstream the Recognition Patch

Previous studies have reported that residues S61 and S64 are phosphorylation sites of mouse Bid, with S61 being more crucial than S64 for regulating apoptosis via phosphorylation.^24^ To verify the significance of these phosphorylation sites in our cleavage assay, we performed assays using phosphomimetic mutations S61E and S64E (Figure 2C). Both variants resulted in significant reductions (Δ*k* < −45%) in the cleavage rate, confirming the biological relevance of the present Caspase-8-mediated cleavage assay in Bcl-2-regulated apoptosis. Additionally, we prepared single-mutation variants S61Q and S64Q, which mimic the steric hindrance of phosphoserine while maintaining a neutral charge (Figure 2C). These variants also caused reductions in the cleavage rate (Δ*k* < −30%), but the effects were less pronounced compared to the phosphomimetic mutations S61E and S64E. This result suggests that both charge and steric hindrance impact cleavage efficiency.

Another notable finding from the alanine-scanning study is that the 61AA variant (S61A-Q62A) significantly increases the cleavage rate (Δ*k* > 46%; Figure 1C). To investigate the molecular basis for this enhancement, we conducted molecular dynamics (MD) simulations (discussed in a later section) and generated single-mutation variants to modify the side-chain properties. Both the S61A and S61G mutations led to a marked increase in cleavage rate (Figures 2D and S2), with S61A and S61G enhancing cleavage by over 15% and 25%, respectively. This trend suggests that reducing the side-chain size at position 61 progressively enhances cleavage efficiency, indicating that a smaller side chain at this site facilitates the interaction with Caspase-8 and promotes cleavage.

The Q62A and Q62G variants had minimal effect on the cleavage rate, suggesting that site Q62 is not as critical as S61. However, when both residues were mutated to alanine, the 61AA variant significantly enhanced the cleavage rate, indicating a positive epistasis.

## MD Simulations

We conducted MD simulations to investigate the structural dynamics of WT Bid and its variants. Geometric parameters, including root-mean-square deviation (RMSD) and radius of gyration (*R*_g_), were employed to assess system stability and simulation convergence (Figure S3). Analysis of per-residue root-mean-square fluctuation (RMSF) during the equilibrated period (>400 ns) (Figure 3A), along with corresponding cartoon illustrations, revealed differences in dynamical fluctuations (or structural flexibility) between WT Bid and the 61AA variant (ΔRMSF in Figure 3B).

**Figure 3:**
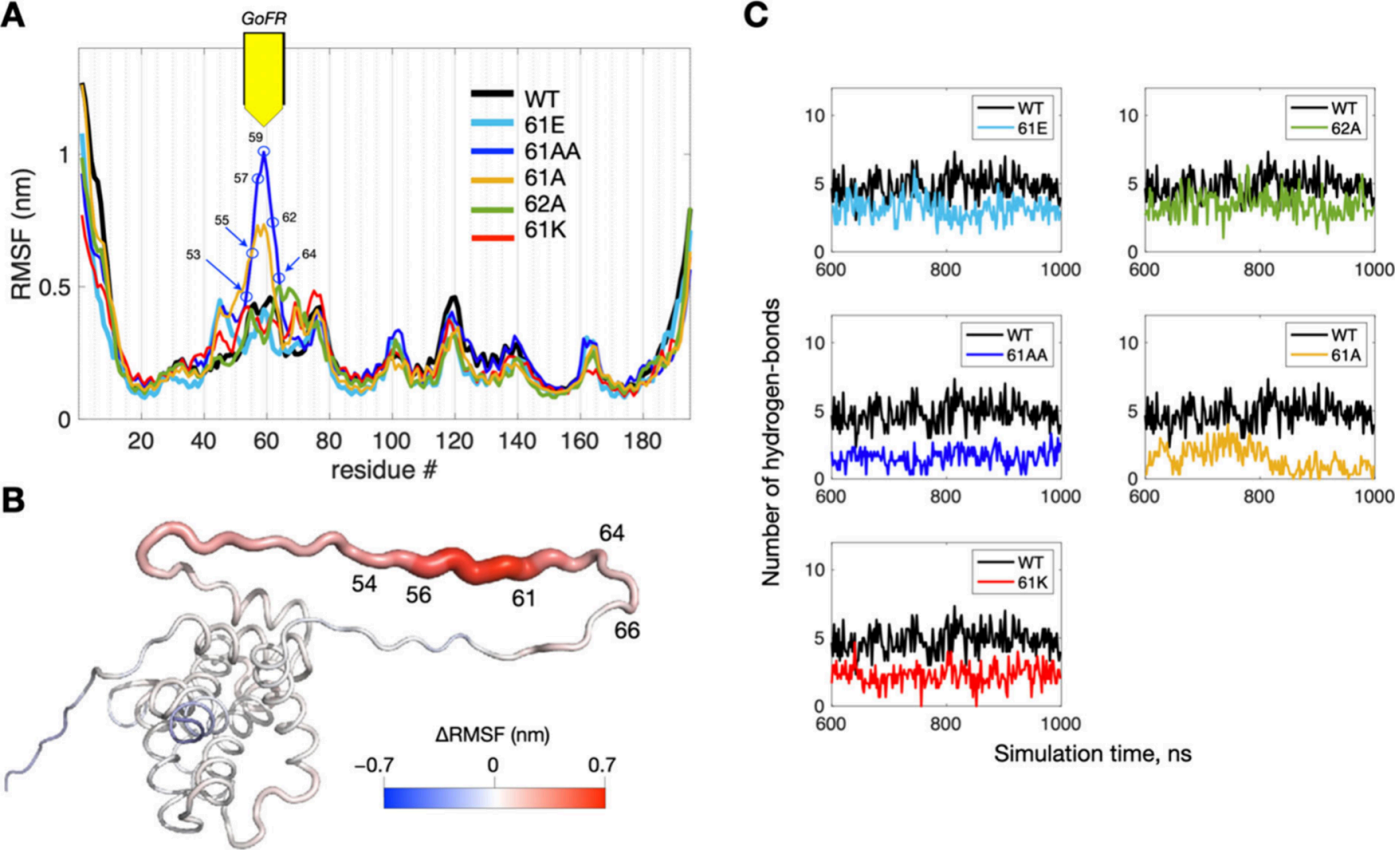
MD Simulations. (A) Per-residue RMSF results from the MD simulations of various Bid variants, highlighting the GoFR sequence (53–64). This region corresponds to the gain-of-flexibility observed between the 61AA variant and other Bid variants including WT Bid. RMSF in the GoFR is clearly increased in the 61AA variant. (B) Cartoon structural model illustrating the ΔRMSF results, representing the differences in RMSF between WT and 61AA. The ΔRMSF data were projected onto the selected representative structures, using the ribbon thickness and color to show ΔRMSF values. (C) Number of hydrogen bonds observed for the indicated Bid variants during the equilibrated simulation period from 600 to 1000 ns. See also Figure S3 for additional MD results.

The RMSF results revealed a significant increase in structural flexibility of residues 53–64 (Figure 3A) when both S61 and Q62 were mutated to alanine, compared to the WT. This flexibility increased moderately when only S61 was mutated to alanine. Consequently, these residues, 53–64, are referred to as the gain-of-flexibility region (GoFR) hereafter. Other mutations at residues 61 and 62, such as 61E, 62A, and 61K, showed only subtle differences in RMSF compared to the WT.

Further analysis of the MD simulations provided deeper insights into the molecular details. We plotted the total number of hydrogen bonds within GoFR over the equilibrated simulation time from 600 to 1000 ns (Figure 3C). The average number of hydrogen bonds within GoFR in the Bid variants was similar to that of WT (∼4.8), except in the 61A and 61AA variants. Notably, the 61AA variant exhibited a significantly lower average number of hydrogen bonds (∼1.58) than the others. The 61AA mutation appears to substantially disrupt hydrogen bond formation within GoFR, consequently increasing its flexibility. The MD simulations support the conclusion that the reduced number of hydrogen bonds within GoFR is the primary factor contributing to the increased flexibility observed with the 61AA mutation.

To identify which interactions were removed or added by the mutations and how these changes affect the flexibility of GoFR, we analyzed the MD simulation results to extract the top residues contributing to hydrogen bonding. In the simulations for WT, residue pairs R71-E55 and R40-D54 exhibited the highest occupancy (>90%), indicating that they are frequently formed and likely play an important role in the structure and function of the loop. In contrast, the simulations for the 61AA variant showed that all hydrogen bonds identified throughout the MD trajectory had low occupancy (<10%).

Although residues S61 and Q62 can potentially contribute to hydrogen bonding, their occupancy values were very low (<10%) throughout all MD trajectories of WT performed (three replicas). This suggests that hydrogen bonds involving S61 and Q62 are infrequent and do not play a significant role in the structure or flexibility of the loop in WT. Consequently, we conclude that S61 and Q62 do not directly contribute to the observed rigidity of GoFR through hydrogen bond formation in WT.

These results reveal a previously unrecognized allosteric pathway that links hydrogen-bonding in the GoFR to Bid cleavage. Substituting S61 and Q62 with alanine (61AA) removes key donors/acceptors and reduces the average number of GoFR-core hydrogen-bonds from ∼4.8 to ∼1.6. Freed from these constraints, the loop samples wider conformations, allowing the 53–60 recognition patch to engage Caspase-8 more efficiently and thereby accelerate proteolysis. A quasi-harmonic analysis of the equilibrated trajectories supports this view:^25^ the entropic free-energy term (T·S) for the GoFR is 5.6 ± 1.9 kJ mol^–1^ residue^–1^ higher in 61AA than in WT at 300 K (see SI Methods). We therefore propose that adding (or reinstating) specific hydrogen-bond contacts tunes local loop entropy and offers a practical handle for modulating Caspase-8 selectivity without altering the enzyme’s catalytic site.

Additionally, we performed MD simulations for the 53AAA variant and analyzed its RMSF profile to assess loop dynamics (Figure S3C). The RMSF values showed only minor changes between WT and 53AAA, indicating that, unlike the 61AA variant, alanine substitutions at residues 53–55 did not significantly alter loop flexibility. This finding suggests that the 53AAA mutations do not allosterically influence loop dynamics. Instead, the observed reduction with the 53–55 variants in cleavage efficiency in the proteolysis assay can be attributed to disrupted substrate recognition rather than changes in loop flexibility (Figure 2). These results support our conclusion that the E53-D54-E55 residues play a critical role in substrate recognition, reinforcing the importance of the E53-D54-E55 motif in determining the Bid-caspase-8 interaction.

It is worth emphasizing that our investigation of loop flexibility arose from the idea that, although the Bid loop is dynamic, it is not entirely unstructured. Instead, it retains partial structural elements that are vital for its function. Indeed, our MD simulations revealed transient formation and dissipation of small structured segments within the loop, reinforcing this partially ordered model. If the loop were fully disordered, further increases in flexibility would have minimal influence on substrate recognition. Consequently, the pronounced flexibility observed in the GoFR region gains significance because this partially structured loop can adopt conformations more favorable for Caspase-8 binding.

## Conformational Analysis of the Bid Loop by DEER Spectroscopy

To probe Bid-loop dynamics experimentally, we performed four-pulse DEER measurements on carefully designed spin-labeled variants. We chose two mutants expected to represent the extremes of GoFR flexibility: 61E, a phosphomimetic that greatly slows Caspase-8 cleavage, and 61AA, a gain-of-function mutant that accelerates cleavage. Because our proteolysis assays showed that even a single substitution inside the GoFR (residues 53–64) can markedly alter loop behavior or disrupt Caspase-8 recognition, we deliberately avoided placing nitroxide labels within the GoFR itself. Inserting a spin label directly into the regulatory segment would risk masking the native conformational changes we wished to measure.

Instead, we selected two loop positions, 44 and 50, that flank, but do not penetrate, the GoFR. Each of these sites was paired with a second label in a well-structured region of Bid (positions 30, 82, or 154) (Figure 4A). This labeling strategy allows us to sense GoFR motions while minimizing local perturbation of the region under study.

**Figure 4:**
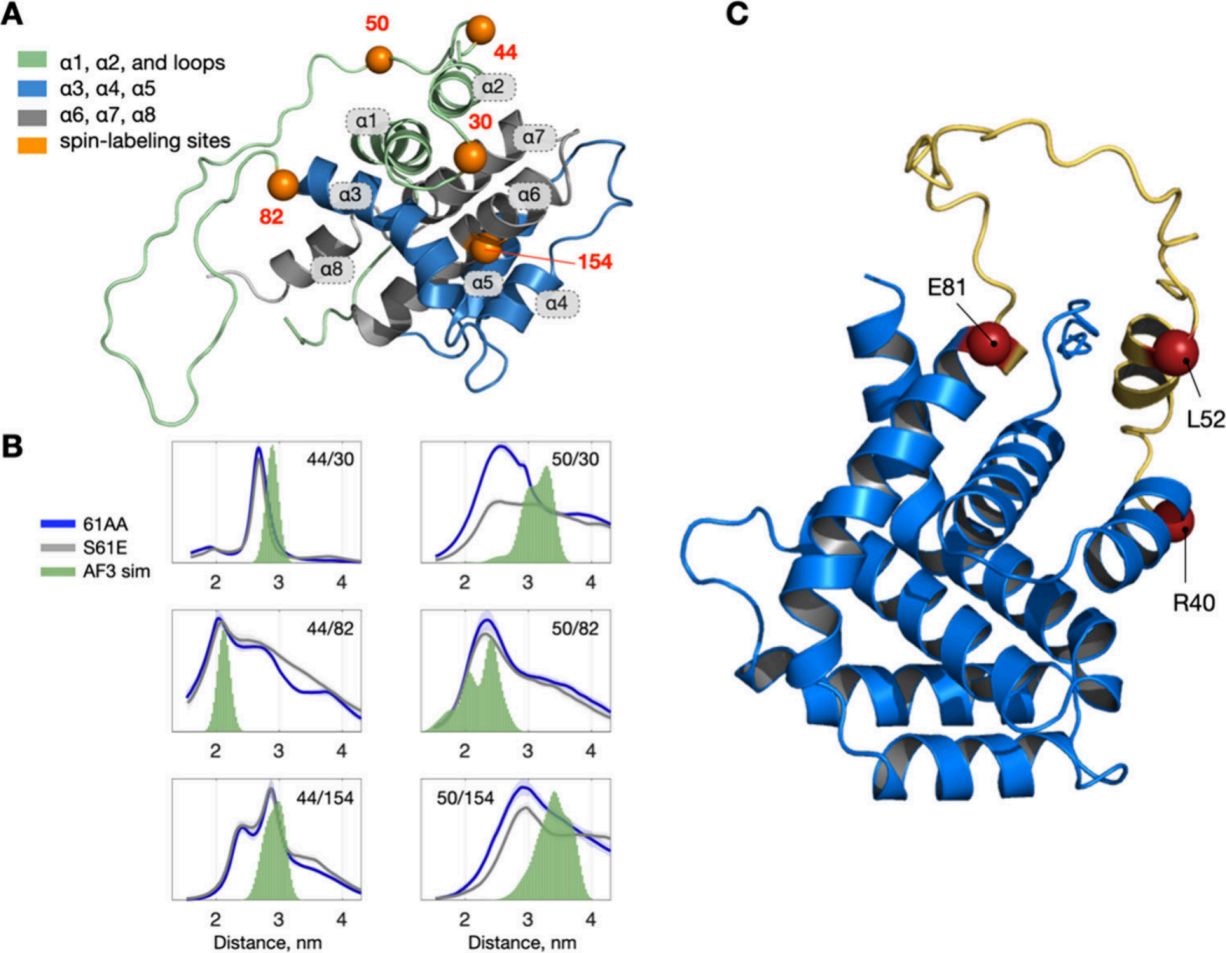
DEER Measurements. (A) Structural model of Bid (PDB: 1DDB; see also Figure S4C for all NMR models) showing the mutation sites used in the spin-labeling study. (B) Distance distributions for double-labeled Bid variants 61AA and S61E. Spin-labeling sites are indicated in each plot. Shaded areas represent the 95% confidence interval. The 61AA variant represents the gain-of-flexibility mutation, while S61E is a phosphomimetic variant. See also Figure S4A for experimental DEER traces. Simulations based on the AF3 model of WT Bid align reasonably with our DEER results. (C) AF3 model of WT Bid showing part of Bid’s 42-residue loop (residues 40–81; yellow) may adopt a short helical segment (residues 46–52), although the associated confidence scores (plDDT < 50) of the loop are lower than those for the rest of the protein. These results suggest that this loop retains some structural order, contrasting with the earlier NMR model, which indicated a fully disordered region.

Distance distributions (Figure 4B) extracted from the DEER time traces (Figure S4) support the computational predictions. When the loop label was at position 44, spin-labeled variants 61E and 61AA produced nearly identical distance profiles, consistent with limited GoFR sensitivity at that location. By contrast, labeling at position 50, which is directly adjacent to the GoFR, revealed clear differences in the distance distribution between the two mutants. Together with our MD data, these results not only demonstrate our allosteric model but also confirm that off-GoFR labeling avoids artifactual perturbation.

A comparison with the existing NMR structure of Bid (Figure S4C) is complicated by that model’s extremely disordered loop, which spans 20 conformers and precludes meaningful simulation-based comparisons with the DEER data. In contrast, the AlphaFold-3 (AF3) prediction of WT Bid (Figure 4C) reveals a short helical segment within the loop, indicating a partially ordered conformation more in line with our observed distance distributions. To validate this computational model, we simulated interspin distances using the AF3 structure and found them to be reasonably consistent with our experimental DEER results (Figure 4B). This suggests that, under our experimental conditions, the Bid loop retains a degree of structural organization not captured in the highly disordered NMR-derived ensemble. Moreover, our MD simulations point to key electrostatic interactions and hydrogen bonding that may stabilize the loop, supporting the AF3-based perspective. While neither approach completely encompasses the full range of possible conformations, the consistency between AF3 predictions, DEER measurements, and MD simulations underscores the importance of integrating computational and experimental methods to characterize the dynamic structural landscape of Bid.

## AlphaFold Modeling Unveils Bid Loop Structure and Active Site Arrangement

To gain deeper insights into how Caspase-8 cleaves Bid, we also employed AF3 modeling to predict the complex formed by the active Caspase-8 heterotetramer (p18 and p10 subunits) and Bid.^26^ The AF3 model revealed that a portion of Bid’s 42-residue loop adopts a secondary helical structure (Figure S5). In the AF3-derived complex, Bid’s recognition motif engages the p18 subunit’s active site, placing the catalytic dyad (H317 and C360) near the D59–G60 cleavage site (Figures 5A and 5B), supporting the validity of our docking model.

**Figure 5:**
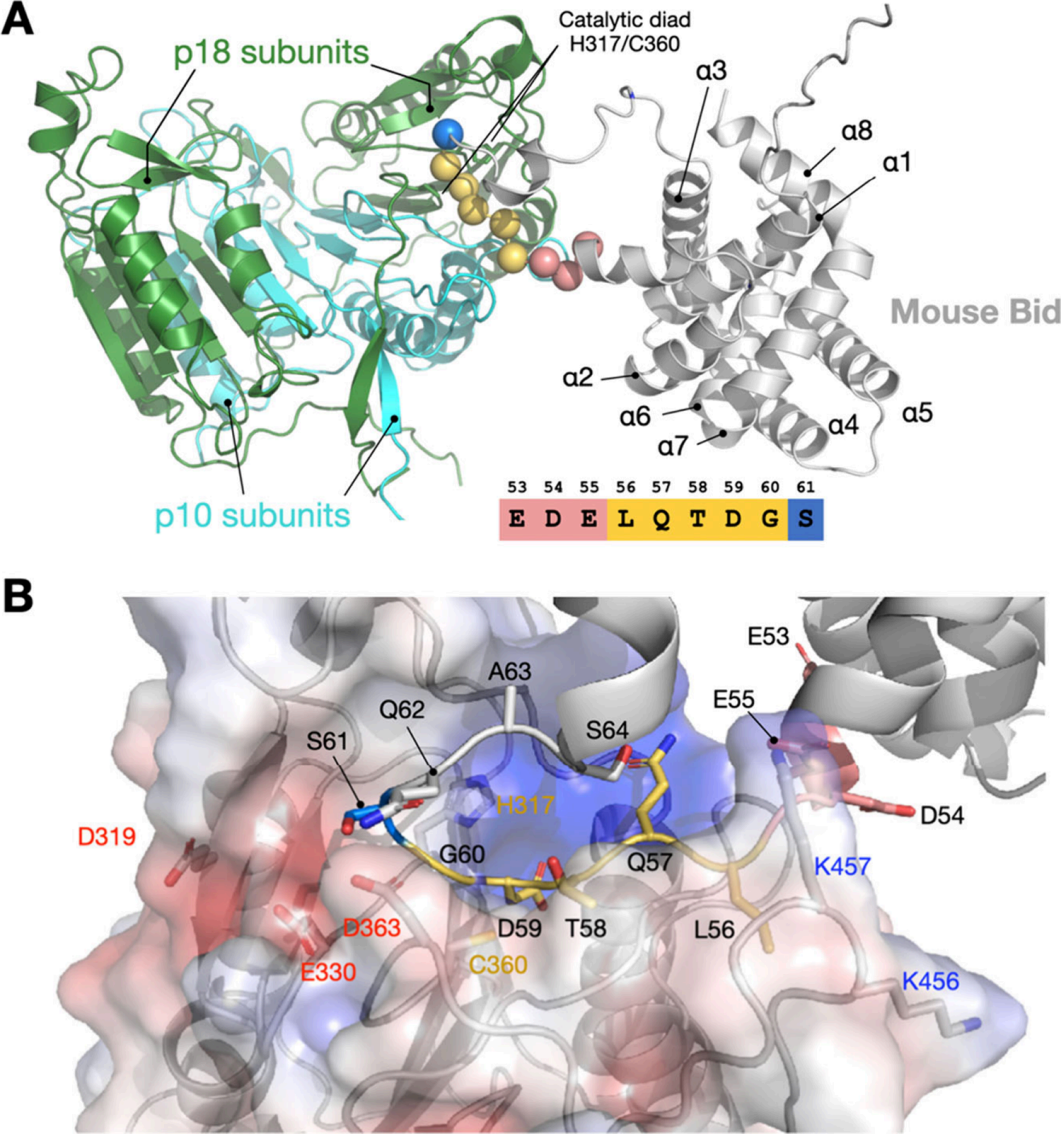
AlphaFold-3 model of the Bid and Caspase-8 complex. (A) AF3 structural model of the Caspase-8 heterotetramer (comprising two p18 and two p10 subunits) in complex with Bid. The 53–61 residues are displayed as balls and colored according to the inset. (B) Electrostatic surface potential of the Caspase-8 active site. Positive charges are shown in blue and negative charges in red. Bid’s loop region is depicted as a ribbon, with side chains of residues 53–64 displayed. The canonical recognition motif is colored yellow, with negatively charged residues (53–55) in red, and the regulatory residue S61 highlighted in blue. Bid residue numbers are labeled in black, while Caspase-8 residue numbers are marked in corresponding colors (the catalytic dyad is shown in yellow, and charged residues are indicated in red and blue). See also Figure S5 for additional results.

To further probe the Bid–Caspase-8 interaction, we generated an electrostatic potential surface map of Caspase-8 (Figures 5B and S5). The analysis suggests that Bid’s negatively charged residues E53, D54, and E55 form stabilizing electrostatic interactions with positively charged K456 or K457 on Caspase-8. Supporting this, mutating these acidic residues to nonpolar (Ala) or polar but uncharged amino acids (Gln or Ser) disrupted cleavage kinetics (Figures 2A and 2B).

Residues G60 and S61, corresponding to P1′ and P2′ (the first and second amino acid positions immediately downstream of the cleavage site) sites on Bid, lie near a negatively charged surface of Caspase-8. Introducing hydrophobic alanine at these positions weakens binding and reduces cleavage rates. Indeed, G60A significantly decreased cleavage efficiency (Figure 2C), aligning with previous phage display data by Agard et al.,^27^ which found that Caspase-8 favors small, polar residues (Gly or Ser) at the P1′ site. In contrast, S61A increased cleavage efficiency by enhancing loop flexibility (Figure 2D). MD simulations confirm that the 61A and 61AA mutations reduce loop-associated hydrogen bonding, increasing flexibility and promoting more efficient proteolysis. Thus, while G60 contributes to favorable substrate interactions, S61 modulates loop dynamics critical for effective enzyme–substrate recognition.

Phosphorylation is a known regulatory mechanism that reduces Bid’s pro-apoptotic function by inhibiting its cleavage.^24,28^ Desagher et al. demonstrated that T58, S61, and S64 in mouse Bid are phosphorylated by casein kinases I and II, protecting Bid from cleavage.^24^ In our study, introducing phosphomimetic mutations S61E and S64E significantly lowered Bid cleavage rates (Figure 2C), validating the inhibitory role of phosphorylation. The AF3 model (Figure 5B) suggests that phosphorylating S61 introduces additional negative charge, creating electrostatic repulsion with Caspase-8 residues D319, E330, and E363. Similar effects at T58 or S64 could also destabilize the substrate and hinder docking at the active site.

Overall, the AF3 predictions and our experimental data indicate that phosphorylation at residues downstream of the cleavage site can regulate cleavage rates by destabilizing substrate–enzyme interactions through electrostatic repulsion. This mechanism provides a molecular basis for how phosphorylation can modulate Bid’s susceptibility to cleavage by Caspase-8, thereby influencing its pro-apoptotic activity.

In sum, Bid recognition is governed by two physical modules: a static charge latch (53–55) that places the LQTD motif in the active-site cleft, and a dynamic entropy hinge (61–62) that decides whether catalysis proceeds. Neutralising the latch slows cleavage by >40%, whereas releasing the hinge boosts activity by ∼50%, effects that correlate with electrostatic repulsion and loop fluctuations measured by DEER. The resulting clamp–hinge model explains how apparently unstructured regions encode precise enzymatic control and offers a general framework for tuning protease selectivity through distributed electrostatics and conformational entropy. Such principles should aid the design of small molecules or peptides that reprogram the Bid–Caspase-8 axis or analogous death-pathway checkpoints.

## Supplementary Material



## Data Availability

All data needed to evaluate the conclusions in the paper are present in the paper and/or the Supporting Information.

## Notes

SI Methods and Figures: Figures S1 and S2 show the proteolysis assays and analysis results; Figure S3 presents supplementary data from MD simulations; Figure S4 shows DEER data and NMR models; Figure S5 shows the AlphaFold-3 model of Bid docked with Caspase-8 (PDF)

## References

[1] Westermann B. (2010). Mitochondrial
Fusion and Fission in Cell Life and Death. Nat.
Rev. Mol. Cell Biol..

[2] Bock F. J., Tait S. W. G. (2020). Mitochondria
as Multifaceted Regulators of Cell Death. Nat.
Rev. Mol. Cell Biol..

[3] Czabotar P. E., Lessene G., Strasser A., Adams J. M. (2014). Control
of Apoptosis
by the BCL-2 Protein Family: Implications for Physiology and Therapy. Nature reviews. Molecular cell biology.

[4] King L. E., Hohorst L., García-Sáez A. J. (2023). Expanding
Roles
of BCL-2 Proteins in Apoptosis Execution and Beyond. Journal of Cell Science.

[5] Kantari C., Walczak H. (2011). Caspase-8 and Bid:
Caught in the Act between Death
Receptors and Mitochondria. Biochimica et Biophysica
Acta (BBA) - Molecular Cell Research.

[6] Timmer J. C., Salvesen G. S. (2007). Caspase Substrates. Cell Death
Differ..

[7] Oh K. J., Barbuto S., Meyer N., Kim R.-S., Collier R. J., Korsmeyer S. J. (2005). Conformational Changes in BID, a pro-Apoptotic BCL-2
Family Member, upon Membrane Binding. A Site-Directed Spin Labeling
Study. J. Biol. Chem..

[8] Shamas-Din A., Bindner S., Zhu W., Zaltsman Y., Campbell C., Gross A., Leber B., Andrews D. W., Fradin C. (2013). tBid Undergoes
Multiple Conformational Changes at the Membrane Required for Bax Activation. J. Biol. Chem..

[9] Wang Y., Tjandra N. (2013). Structural Insights
of tBid, the Caspase-8-Activated
Bid, and Its BH3domain. J. Biol. Chem..

[10] Hung C.-L., Chang H.-H., Lee S. W., Chiang Y.-W. (2021). Stepwise Activation
of the Pro-Apoptotic Protein Bid at Mitochondrial Membranes. Cell Death Differ..

[11] Chou J. J., Li H., Salvesen G. S., Yuan J., Wagner G. (1999). Solution Structure
of BID, an Intracellular Amplifier of Apoptotic Signaling. Cell.

[12] McDonnell J. M., Fushman D., Milliman C. L., Korsmeyer S. J., Cowburn D. (1999). Solution Structure of the Proapoptotic Molecule BID:
A Structural Basis for Apoptotic Agonists and Antagonists. Cell.

[13] Hung C.-L., Lin Y.-Y., Chang H.-H., Chiang Y.-W. (2018). Accessing
Local
Structural Disruption of Bid Protein during Thermal Denaturation by
Absorption-Mode ESR Spectroscopy. RSC Adv..

[14] Hung C.-L., Kuo Y.-H., Lee S. W., Chiang Y.-W. (2021). Protein Stability
Depends Critically on the Surface Hydrogen-Bonding Network: A Case
Study of Bid Protein. J. Phys. Chem. B.

[15] Priya P., Maity A., Ghosh Dastidar S. (2017). The Long Unstructured Region of Bcl-xl
Modulates Its Structural Dynamics. Proteins.

[16] Rogers J.
M., Wong C. T., Clarke J. (2014). Coupled Folding and Binding of the
Disordered Protein PUMA Does Not Require Particular Residual Structure. J. Am. Chem. Soc..

[17] Lan Y.-J., Yeh P.-S., Kao T.-Y., Lo Y.-C., Sue S.-C., Chen Y.-W., Hwang D. W., Chiang Y.-W. (2020). Anti-Apoptotic BCL-2
Regulation by Changes in Dynamics of Its Long Unstructured Loop. Commun. Biol..

[18] Dengler M. A., Robin A. Y., Gibson L., Li M. X., Sandow J. J., Iyer S., Webb A. I., Westphal D., Dewson G., Adams J. M. (2019). BAX Activation: Mutations Near Its
Proposed Non-Canonical
BH3 Binding Site Reveal Allosteric Changes Controlling Mitochondrial
Association. Cell Reports.

[19] Morrison K. L., Weiss G. A. (2001). Combinatorial Alanine-Scanning. Curr. Opin. Chem. Biol..

[20] Schiemann O., Heubach C. A., Abdullin D., Ackermann K., Azarkh M., Bagryanskaya E. G., Drescher M., Endeward B., Freed J. H., Galazzo L., Goldfarb D., Hett T., Esteban Hofer L., Fábregas Ibáñez L., Hustedt E. J., Kucher S., Kuprov I., Lovett J. E., Meyer A., Ruthstein S., Saxena S., Stoll S., Timmel C. R., Di Valentin M., Mchaourab H. S., Prisner T. F., Bode B. E., Bordignon E., Bennati M., Jeschke G. (2021). Benchmark Test and Guidelines for
DEER/PELDOR Experiments on Nitroxide-Labeled Biomolecules. J. Am. Chem. Soc..

[21] Yeh P.-S., Li C.-C., Lu Y.-S., Chiang Y.-W. (2023). Structural Insights
into the Binding and Degradation Mechanisms of Protoporphyrin IX by
the Translocator Protein TSPO. JACS Au.

[22] Li C.-C., Kao T.-Y., Cheng C.-C., Chiang Y.-W. (2020). Structure and Regulation
of the BsYetJ Calcium Channel in Lipid Nanodiscs. Proc. Natl. Acad. Sci. U. S. A..

[23] Galazzo L., Meier G., Januliene D., Parey K., De Vecchis D., Striednig B., Hilbi H., Schäfer L. V., Kuprov I., Moeller A., Bordignon E., Seeger M. A. (2022). The ABC Transporter MsbA Adopts the
Wide Inward-Open
Conformation in *E. Coli* Cells. Sci. Adv..

[24] Desagher S., Osen-Sand A., Montessuit S., Magnenat E., Vilbois F., Hochmann A., Journot L., Antonsson B., Martinou J.-C. (2001). Phosphorylation of Bid by Casein Kinases I and II Regulates
Its Cleavage by Caspase 8. Mol. Cell.

[25] Baron R., Hünenberger P. H., McCammon J. A. (2009). Absolute Single-Molecule
Entropies from Quasi-Harmonic Analysis of Microsecond Molecular Dynamics:
Correction Terms and Convergence Properties. J. Chem. Theory Comput..

[26] Abramson J., Adler J., Dunger J., Evans R., Green T., Pritzel A., Ronneberger O., Willmore L., Ballard A. J., Bambrick J., Bodenstein S. W., Evans D. A., Hung C.-C., O’Neill M., Reiman D., Tunyasuvunakool K., Wu Z., Žemgulytė A., Arvaniti E., Beattie C., Bertolli O., Bridgland A., Cherepanov A., Congreve M., Cowen-Rivers A. I., Cowie A., Figurnov M., Fuchs F. B., Gladman H., Jain R., Khan Y. A., Low C. M. R., Perlin K., Potapenko A., Savy P., Singh S., Stecula A., Thillaisundaram A., Tong C., Yakneen S., Zhong E. D., Zielinski M., Žídek A., Bapst V., Kohli P., Jaderberg M., Hassabis D., Jumper J. M. (2024). Accurate Structure
Prediction of
Biomolecular Interactions with AlphaFold 3. Nature.

[27] Agard N. J., Mahrus S., Trinidad J. C., Lynn A., Burlingame A. L., Wells J. A. (2012). Global Kinetic Analysis of Proteolysis
via Quantitative
Targeted Proteomics. Proc. Natl. Acad. Sci.
U.S.A..

[28] Esposti M. D., Ferry G., Masdehors P., Boutin J. A., Hickman J. A., Dive C. (2003). Post-Translational Modification of Bid Has Differential Effects on
Its Susceptibility to Cleavage by Caspase 8 or Caspase 3. J. Biol. Chem..

