# Supplementary material for: Electrostatic Clamp and Loop Dynamics Dictate Caspase‑8 Cleavage of the Apoptotic Protein Bid
Source: J Phys Chem Lett. 2025 Jul 18;16(30):7522–9. doi: 10.1021/acs.jpclett.5c01976 (PMC12319900; doi:10.1021/acs.jpclett.5c01976)
Supplement: Supplementary file 1 [file jz5c01976_si_001.pdf]

Supporting Information for

Electrostatic Clamp and Loop Dynamics Dictate Caspase-8 Cleavage of the Apoptotic Protein Bid

Chien-Lun Hung,[a] Wen-Hsien Wang,[a] Yu-Chuan Chang,[a] Yei-Chen Lai,*[b] and Yun-Wei Chiang*[a]

[a]Department of Chemistry, National Tsing Hua University, Hsinchu 300-044, Taiwan

[b]Department of Chemistry, National Chung Hsing University, Taichung 402-202, Taiwan

*Corresponding email: yeichenlai@nchu.edu.tw, ywchiang@mx.nthu.edu.tw

SI MATERIALS AND METHODS

Bid Expression and Purification

Full-length mouse Bid was cloned into NdeI/XhoI site of pET28a vector (New England Biolabs, Inc.). WT Bid has two native cysteine residues (C30 and C126). Cysteine-free construct (C30S/C126S) was used to prepare cysteine variants of Bid for spin-labeling and DEER studies. Point mutations of recombinant Bid were generated using the QuikChange site-directed mutagenesis kit (Stratagene) and verified by DNA sequencing. The recombinant pET28a vector was transformed into the E. coli BL21(DE3) expression strain (Novagen). Recombinant proteins fused with six histidines at the N-terminal of Bid were expressed and purified by an affinity Ni-column, as previously described.[1,2] Briefly, bacterial culture was grown at 37 °C in Luria−Bertani (LB) medium containing kanamycin (40 μg/mL) until OD600 reached 0.6–0.8. Protein expression was induced by addition of 1 mM IPTG (isopropyl 1-thio-β-D-galactopyranoside) at 30 °C for 4–6 h. The cell pellet was collected by centrifugation and resuspended in ice-cold lysis buffer (20 mM Tris, pH 7.4, 100 mM NaCl, 20 mM imidazole and 1 mM PMSF). The resuspended pellet was sonicated on ice for 10 min, followed by centrifugation at 25000 g for 30 min. The supernatant was filtrated through a 0.45 μm filter and then loaded onto an affinity Ni column using HisTrap HP (GE Healthcare) at a flow rate about 1 mL/min. The column was washed with 10 column volumes (CV) of wash buffer (50 mM Tris, pH 7.4, 500 mM NaCl and 40 mM imidazole). Bid fraction was eluted with 10 CV of elution buffer (20 mM Tris, pH 7.4, 100 mM NaCl and 500 mM imidazole). Purified protein was confirmed by sodium dodecyl sulfate-polyacrylamide gel electrophoresis (SDS-PAGE) with Coomassie blue staining. Imidazole was removed using a PD-10 desalting column (GE Healthcare) equilibrated with storage buffer (20 mM Tris, pH 7.4, and 100 mM NaCl), and protein concentration was estimated via absorption spectroscopy at 280 nm.

Rationale for Triple-Alanine Scanning

We chose triple-alanine scanning, a widely used form of combinatorial alanine mutagenesis, because it delivers an optimal balance between library size and functional resolution.[3–5] Mutating three adjacent residues at a time reduces the number of constructs by roughly an order of magnitude relative to single-site libraries, yet still uncovers cooperative electrostatic or steric effects that isolated point mutants often miss. Triple-alanine blocks have mapped functional hotspots in a variety of disordered loops, helices and enzyme surfaces—including the BAX activation loop—

demonstrating that this coarse-grained first pass reliably highlights "hot regions" for subsequent single- or double-site dissection.[6] Consistent with that strategy, our initial triple scan flagged the 53–55 and 61–62 segments, after which targeted single and double mutants clarified the individual contributions of each residue.

Caspase-8 Expression and Purification

The truncated human Caspase-8 (ΔDEDs-Casp-8, amino acids 217–496) was cloned into NdeI/BamHI site of pET15b vector (New England Biolabs, Inc.), as previously reported.[1,2] The recombinant pET15b vector was transformed into the E. coli BL21(DE3) pLysS expression strain (Novagen). Recombinant proteins fused with six histidines at the N-terminal of Caspase-8 were expressed and purified by an affinity Ni-column. Bacterial culture was grown at 37 °C in 2xYT medium containing 100 μg/mL ampicillin (Amp) until OD600 reached 0.6–0.8. Protein expression was induced by addition of 0.2 mM of IPTG at 37 °C for 4–6 h. Cells were harvested by centrifugation and resuspended in ice-cold lysis buffer (50 mM Tris, pH 7.4, 100 mM NaCl, 20 mM imidazole, and 1 mM PMSF). The resuspended pellet was sonicated on ice for 10 min, followed by centrifugation at 25000 g for 30 min. The supernatant was filtrated through a 0.45 μm filter and then loaded onto an affinity Ni-column using HisTrap HP (GE Healthcare) at a flow rate about 1 mL/ min. Unbound proteins were removed with 10 CV of wash buffer (50 mM Tris, pH 7.4, 500 mM NaCl and 40 mM imidazole). Caspase-8 fraction was eluted with elution buffer (50 mM Tris, pH 7.4, 100 mM NaCl and 500 mM imidazole). Imidazole was removed using a PD-10 desalting column (GE Healthcare) equilibrated with storage buffer (20 mM Tris, pH 7.4, and 100 mM NaCl). The purified Caspase-8 was confirmed by SDS-PAGE with Coomassie blue staining. Protein concentration was estimated via absorption spectroscopy at 280 nm.

Proteolysis Assay for Bid Cleavage by Caspase-8

Full-length Bid (400 μg, at 2 mg/mL) was incubated with approximately 0.275 mU of Caspase-8 in a storage buffer (20 mM Tris, 100 mM NaCl, pH 7.4) at 25 °C for 1 h. To monitor the cleavage of full-length Bid over time, aliquots were taken every 10 min throughout the 1-h reaction. Each aliquot was immediately mixed with SDS loading dye (250 mM Tris-HCl, 12.5% w/v SDS, 50% v/v glycerol, 5 mM EDTA, 50 μg/mL Bromophenol Blue, 500 mM DTT) and heated at 100 °C for 6 min to halt the reaction.

Samples were then analyzed on a 15% SDS-PAGE gel and stained with Coomassie Blue to visualize the remaining full-length Bid. The band intensities were measured using ImageJ.[7] The percentage of cleavage at each time point was calculated as $(I_0 - I)/I_0 \times 100\%$, where I_0 is the intensity of the full-length Bid band at the start of the reaction, and I is the intensity at a given time point. The time-dependent cleavage profiles were fitted to an exponential equation, $1 - \exp(-kt)$, to determine the reaction rate constant k. For each Bid variant, a control reaction with WT Bid was run in parallel to ensure that the WT reaction rate remained consistent across different experimental batches.

Calibration of Caspase-8 for Consistent Catalytic Efficiency in Time-resolved Proteolysis Assays

To ensure that variations in Caspase-8 catalytic efficiency across different enzyme batches did not introduce inconsistencies into our time-resolved proteolysis assays, we implemented a calibration procedure prior to conducting experiments on Bid cleavage. This calibration aimed to establish a standardized baseline for Caspase-8 activity, allowing us to select an appropriate amount of enzyme that would yield comparable reaction rates between batches.

In this process, a series of reaction mixtures were prepared by combining a fixed concentration of WT Bid with varying concentrations of Caspase-8. Each mixture was then subjected to the standard time-resolved proteolysis protocol: aliquots were removed at defined intervals, quenched immediately with SDS loading dye, and boiled at 100 °C to halt further cleavage. The samples were subsequently analyzed by SDS-PAGE, followed by Coomassie blue staining, to quantify the extent of Bid cleavage.

For each Caspase-8 concentration tested, the cleavage data were fitted to an exponential function to determine the corresponding rate constant (k). By plotting these rate constants against Caspase-8 concentrations, we generated calibration curves that effectively linked enzyme amount to catalytic efficiency. These curves enabled the selection of a Caspase-8 concentration that would produce a desired and consistent reaction rate. Before each new set of time-resolved proteolysis assays, we used these calibration curves to adjust the enzyme amount as necessary, ensuring that

variations in enzyme activity between batches did not compromise the reproducibility and comparability of our results.

MD Simulation and Analysis

The atomistic structure of Bid obtained from UnitProt database (PDB code: 1DDB) was used in the simulation. Variants were modeled using PSFGEN utility of VMD.[8] The systems were soaked in TIP3P water boxes and neutralizing ions (Na^{+} and Cl^{-}) at an experimental concentration (100 mM) using the VMD program. The simulations were performed using the NAMD program with CHARMM36m force field.[9] Periodic boundary conditions, particle mesh Ewald method, SHAKE algorithm for fixing all bonds linking hydrogen atoms, a non-bonded cutoff of 1.2 nm, and a 2-fs time step were used.[10] The systems were heated with Langevin dynamics, using an NPT ensemble, to the simulation temperature 300 K. The solvated proteins were performed minimization with 5000 steps, then run 1000 ns for unbiased molecular dynamics simulation after the equilibration for 3000000 steps in the presence of harmonic constraints acting on the protein at 300 K. Coordinates, energies, and pressures were saved for analysis every 2 ps. The homemade VMD Tcl code, and VMD's plugins were used to analyze RMSD, RMSF about the mean position of Cα atoms, and number of hydrogen bonds formed by loop region.

Hydrogen-bond statistics were extracted with the H-Bonds plug-in in VMD. Trajectories were first stripped of solvent and ions, and the equilibrated segment (600–1000 ns) of each replica was sampled every 2 ns. Two atom selections were defined: (i) *GoFR* — heavy atoms of residues 53–64 (i.e., non-hydrogen atom of the selected residues); (ii) *Protein-minus-GoFR* — heavy atoms of all other residues. The plug-in's default geometric cut-offs (donor–acceptor distance ≤ 3.0 Å; donor–hydrogen–acceptor angle ≥ 160°) were applied. The "all hbonds" flag reports every donor–acceptor pair and its fractional occupancy across the analysed frames. Mean occupancies from three replicas are discussed in the main text.

Quasi-Harmonic Entropy Calculation

Configurational entropy was estimated through quasi-harmonic analysis, a well-established approach that quantifies entropy based on atomic positional fluctuations derived from MD simulations.[11,12] For each system, equilibrated segments of the NAMD trajectories (600–1000 ns)

from three independent replicas were analyzed. Trajectories were initially processed in VMD to perform least-squares fitting of the protein backbone atoms to the first frame, thereby removing global translational and rotational motions. Subsequently, heavy atoms within the GoFR segment (residues 53–64) were selected for further analysis. We computed the mass-weighted covariance matrix for each replica, diagonalised it and converted the eigenvalues to configurational entropies with the quasi-harmonic expression at 300 K.[11,12] Entropy values obtained from each replica were averaged to provide a robust estimate of the configurational entropy for each system. Entropies reported were averaged over replicas.

DEER Measurements and Analysis

Spin-labeling reaction were performed by addition of 10-fold molar excess of MTSSL ((1-Oxyl-2,2,5,5-tetramethylpyrroline-3-methyl) methanethiosulfonate spin label) per cysteine residue into purified Bid protein solution in the dark overnight at 4 °C. Excess MTSSL removal and D_2O buffer (20 mM Tris and 100 mM NaCl, pH 7.4) exchange were performed by centrifuge for several times after MTSSL labeling. For DEER measurements, 30 μL of spin-labeled Bid (0.1 mM, with 30% (v/v) d-glycerol) were transferred into ESR quartz tubes (i.d. 3 mm) and plunge-frozen in liquid nitrogen. DEER experiments were conducted on a Bruker ELEXSYS E580-400 X-band cw/pulse spectrometer with a split-ring resonator (EN4118X-MS3) equipped with a cryogenic ultralow-noise microwave amplifier and a helium gas flow system (4118CF and 4112HV) at 80 K.[13] In this cryoprobe head, spin echo signals are preamplified before entering the microwave bridge, significantly enhancing sensitivity and SNR, thus reducing data acquisition time. A dead-time-free four-pulse constant-time DEER sequence with two-step phase cycling was employed. The observer π and $\pi/2$ pulse durations were 32 ns and 16 ns, respectively, at a frequency approximately 70 MHz lower than the pump pulse. The pump pulse duration was 32 ns and positioned at the maximum of the echo-detected field-swept spectrum. All pulses were amplified using a pulsed traveling wave tube (TWT) amplifier (E580-1030), with data accumulation times ranging from 40 to 60 min. Data analysis was performed using the DEERNet software with default setting, a deep neural network for one-step DEER data analysis.[14]

AlphaFold-3 Prediction of the Bid-Caspase-8 Complex Structure

All AlphaFold-3 (AF3) predictions were performed using the AlphaFold-3 online server.[15] In cells, pro-Caspase-8 undergoes sequential autoproteolytic cleavages starting at Asp374, followed by Asp216 and Asp384. This process generates the large p18 subunit (residues 217–377) and the small p10 subunit (residues 384–479). These subunits assemble into an $\alpha 2\beta 2$ heterotetrameric complex, forming the active Caspase-8 enzyme.[16,17] To predict the structure of the Bid–Caspase-8 complex, we used the full-length sequence of Bid (UniProt: P70444, residues 1–195) along with two copies each of the Caspase-8 p18 (UniProt: Q14790-1) and p10 subunits. The AF3-predicted structure of active Caspase-8 closely resembled the known crystal structure (PDB: 1QTN), confirming a heterotetrameric configuration with high structural similarity. In the AF3 model, the 42-residue loop of Bid was observed to dock into the active site of Caspase-8, suggesting potential interaction sites within the complex. We visualized the AF3-predicted structure and its electrostatic surface potential using PyMOL 3.0.2, providing further insights into the electrostatic properties of the Bid–Caspase-8 interface.

The AF3 structure of Bid was generated from the full-length sequence on the public AlphaFold-3 server (default multimer mode, no template input). The predicted model (pTM = 0.69) was taken without further minimization. According to the AF3 instruction manual, a pTM score above 0.5 means the overall predicted fold for the complex is similar to the true structure. Inter-spin distance distributions were then computed with chiLife (v1.1.6)[18] using its off-rotamer ensemble option: χ_1–χ_4 dihedrals were sampled beyond canonical rotamers (2000 conformers per label), and the resulting coordinates were convoluted to yield the distance distribution curves shown in Figure 4B.

Statistical and Reproducibility

The statistical analysis adopted 2-tailed unpaired student's test to determine p-values. The stars are used to flag level of significance (ns > 0.05; $*p \leq 0.05$; $**p \leq 0.01$; $***p \leq 0.001$). The mean ± standard error (SE) is displayed, unless otherwise stated.

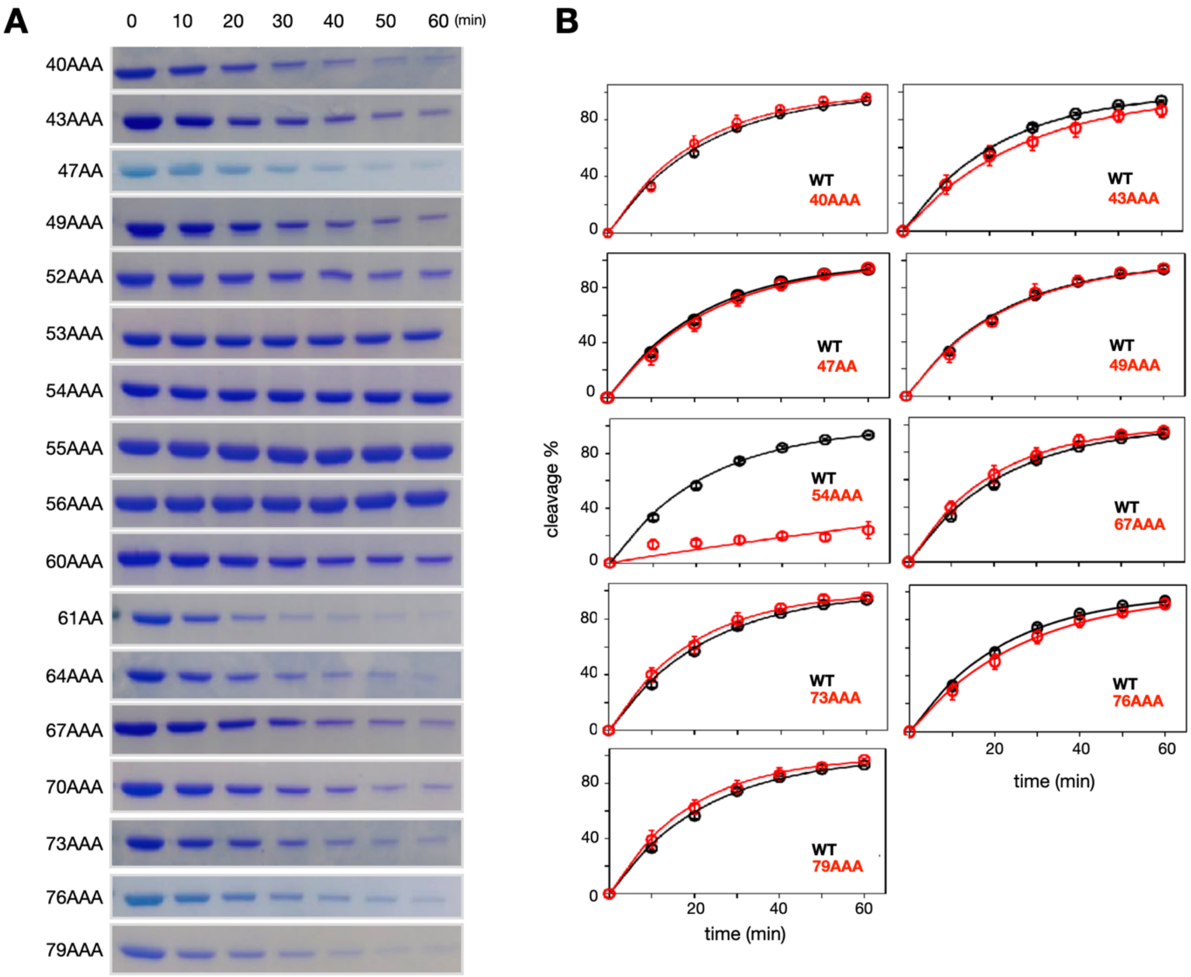

Figure S1. Proteolysis Assays and Analysis Results. (**A**) Representative SDS-PAGE results of the proteolysis assays for various Bid alanine variants, as presented in Figure 1. (**B**) Fitting of the experimental data from the proteolysis assays, used to evaluate the extent of intact Bid cleaved by caspase-8 over time. Some of the fitting results corresponding to the studies displayed in (**A**) are given in Figure 2. Data are presented as mean ± standard error (n>=3 independent experiments).

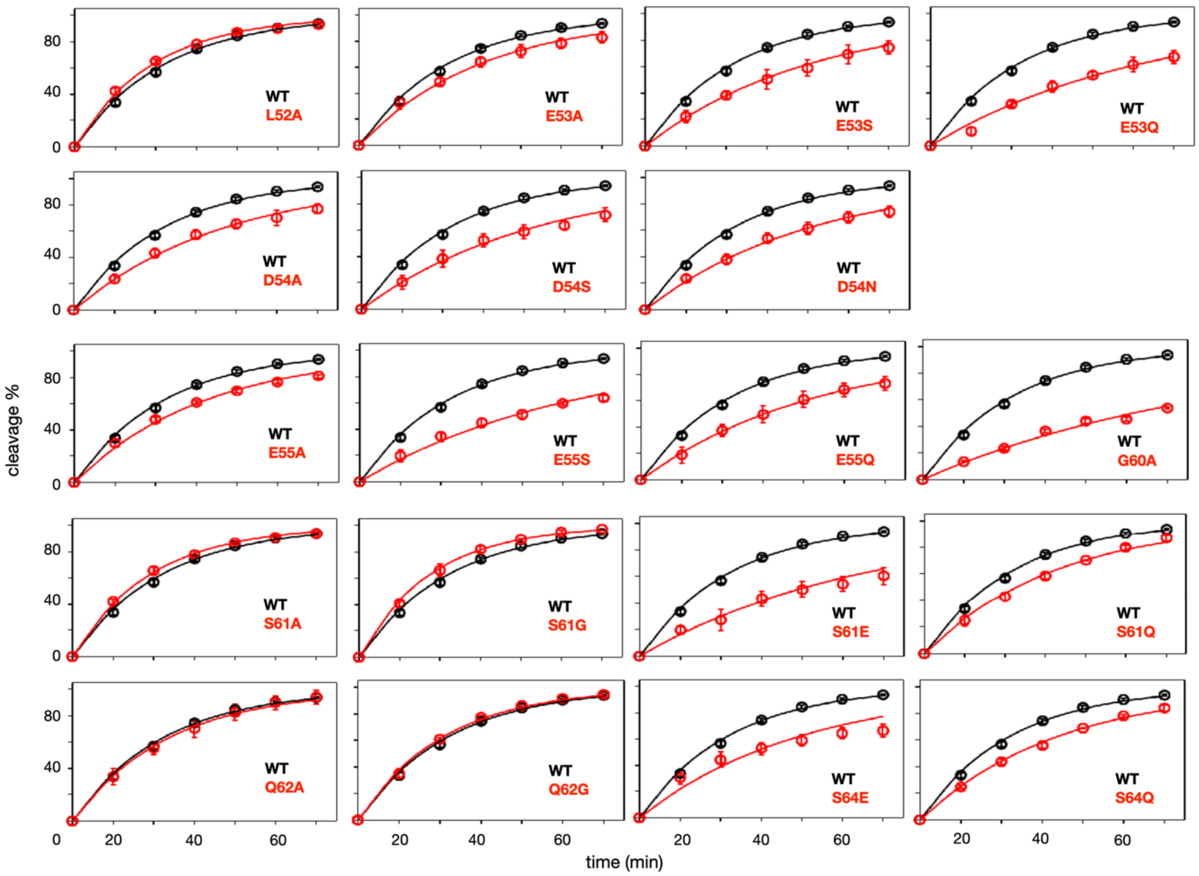

Figure S2. Proteolysis Assays and Analysis Results. The plots shown correspond to the analysis results of the studies presented in Figure 2. They represent the fitting of experimental data from the proteolysis assays, used to evaluate the extent of intact Bid cleaved by caspase-8 over time. Data are presented as mean ± standard error (n>=3 independent experiments).

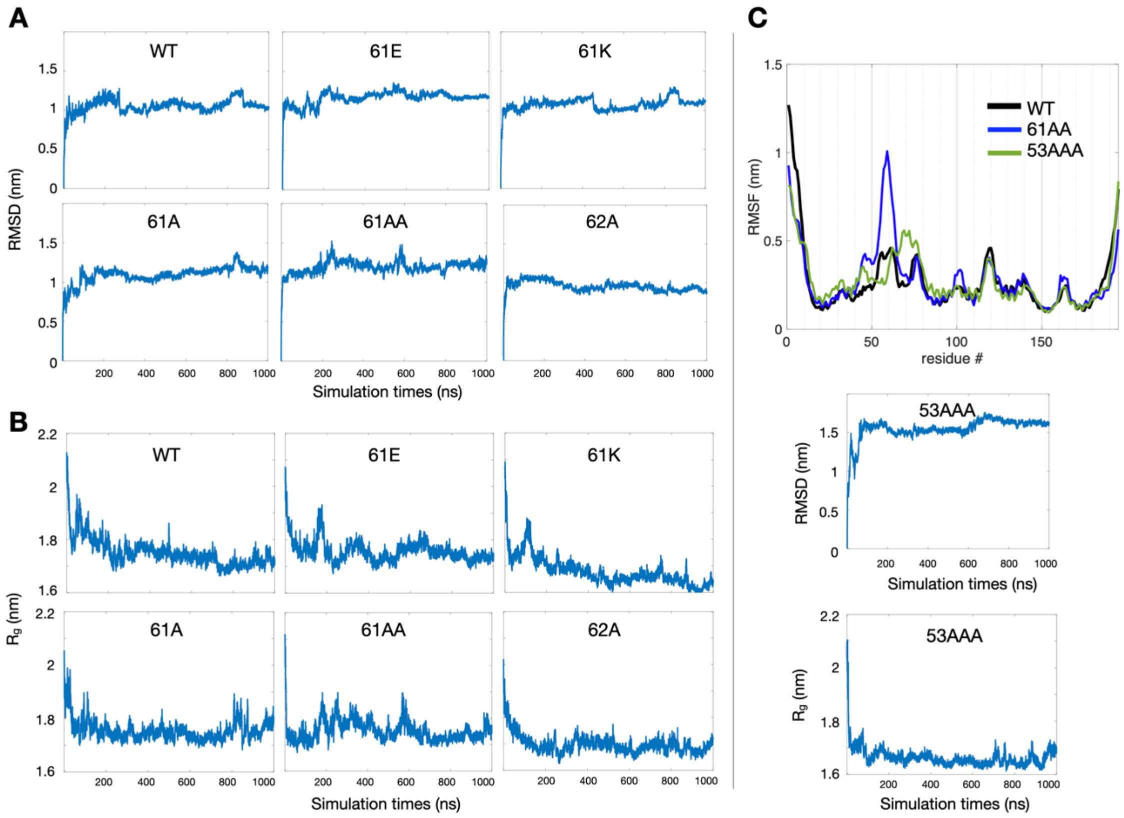

Figure S3. Supplementary Data from MD Simulations. MD simulations were performed in explicit water for 1000 ns with three replicates for each system. (**A**) Representative root mean square deviation (RMSD) and (**B**) radius of gyration (R_g) results for WT Bid and its variants are shown. The RMSD values of all protein atoms were calculated over the entire 1000 ns trajectory, providing an overview of the stability of WT Bid and its variants. The results show that the systems tend to converge after approximately 200 ns, with RMSD values stabilizing around 1 nm for all variants. The R_g values demonstrate good convergence, with the systems stabilizing after ~500 ns and R_g values remaining within 1.68 ± 0.1 nm. Overall, all MD simulations reach convergence around 500 ns and maintain structural stability until the end of the simulation. (**C**) RMSF, RMSD and R_g results for the 53AAA variant. RMSF results for the 53AAA variant are compared with those of WT and 61AA. The loop flexibility of 53AAA is similar to that of WT, as indicated by their comparable RMSF profiles. This suggests that mutating the negatively charged residues E53-D54-E55 to alanine has minimal impact on loop flexibility. These findings support our experimental results, indicating that E53-D54-E55 are more critical for substrate recognition rather than for modulating loop flexibility.

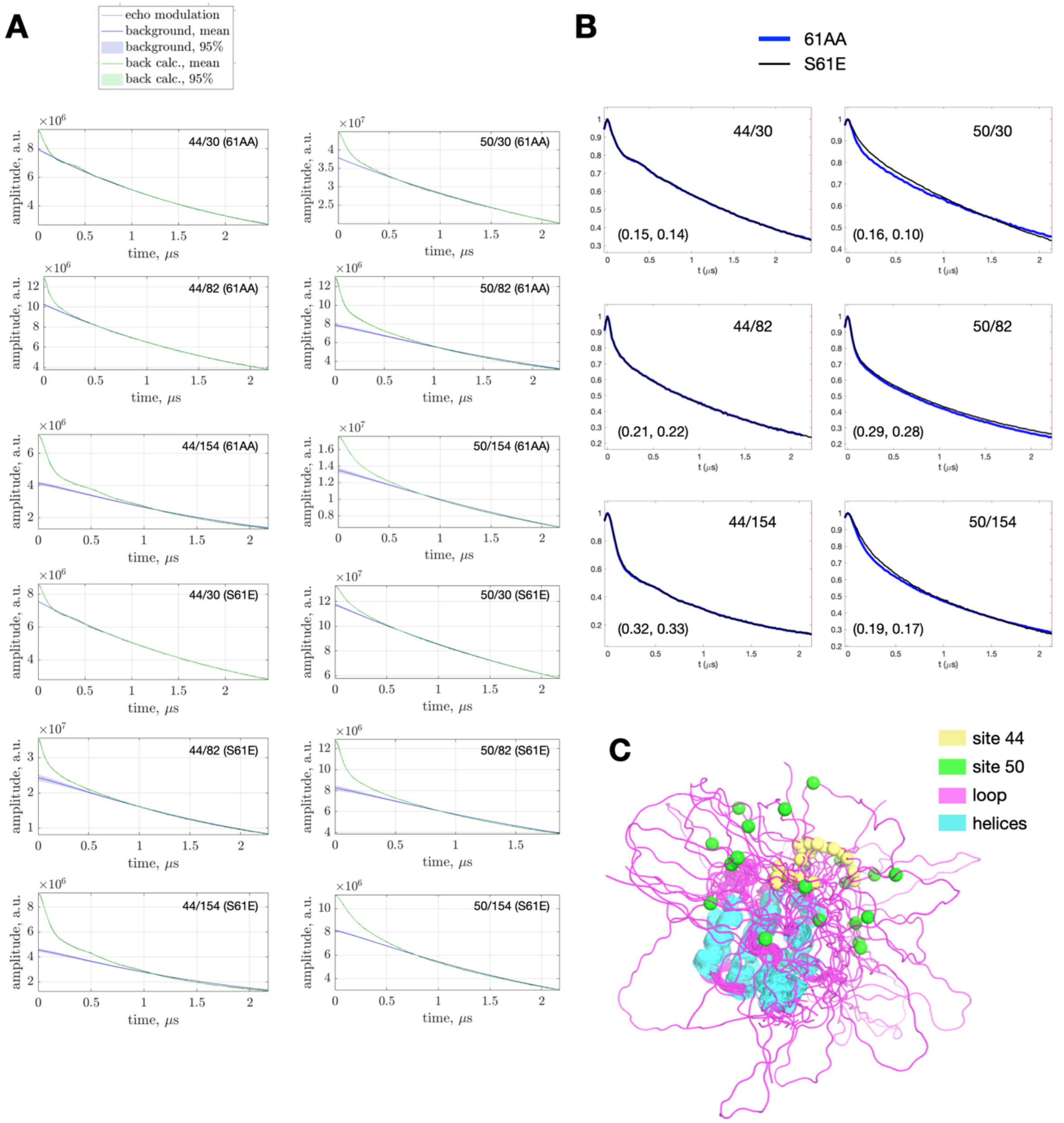

Figure S4. DEER Data and NMR models. (**A**) Raw DEER traces and the DEERNet analysis results. (**B**) Normalized DEER dipolar signals for 61AA (blue) and S61E (black) variants. Modulation depths Δ (61AA, S61E) are given in parentheses on each panel. (**C**) Shown here are the twenty lowest-energy NMR structures of Bid, illustrating the extensive variability of its 42-residue loop (residues 40–81). In these models, the loop appears highly disordered, as evidenced by the broad spatial distribution of spin-labeling sites (positions 44 and 50) used in our DEER experiments. However, our DEER measurements reveal narrower distance distributions,

suggesting that the loop retains partial structural order under our experimental conditions, an observation that contrasts with the fully disordered ensemble inferred from the NMR data. Consequently, we were unable to generate simulated distance distributions consistent with our DEER results from any of the twenty NMR-derived conformers.

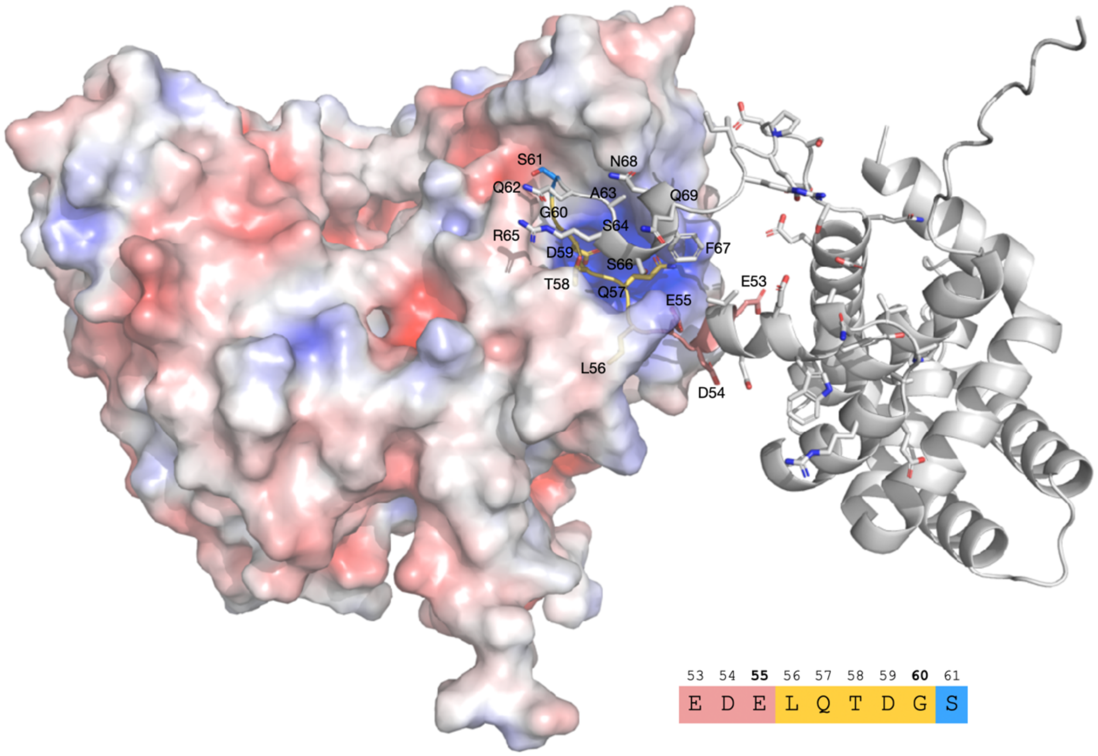

Figure S5. Electrostatic Surface Potential of Caspase-8 Heterotetramer Docked with Bid. The figure depicts an AlphaFold-3 model of Bid (white ribbon) docked with Caspase-8 (surface representation with electrostatic potential). The Caspase-8 active form consists of two p18 subunits and two p10 subunits, and the Bid sequence was submitted separately to AlphaFold 3 for modeling. The predicted structure of Bid aligns well with the known structure (PDB: 1DDB), with only minor differences observed in helix 8, which is slightly bent toward helix 3. Notably, the previously disordered 42-residue loop in Bid is partially structured in the AF3 model of Bid/Caspase-8 bound complex. Residues E53-D54-E55 (red) upstream of the LQTDG cleavage motif (yellow) stabilize the Bid–Caspase-8 binding through electrostatic interactions. In contrast, the S61-Q62-A63 motif (sky blue) is positioned near a negatively charged surface outside the binding pocket, suggesting that phosphorylation at S61 could disrupt the complex and decrease cleavage efficiency. Our experimental data indicate that mutating S61-Q62-A63 to smaller residues promotes complex formation by reducing steric hindrance and increasing flexibility. For reference, the p18 subunit corresponds to residues 217–377 of Caspase-8, and the p10 subunit corresponds to residues 384–479.

SI References

(1) Hung, C.-L.; Chang, H.-H.; Lee, S. W.; Chiang, Y.-W. Stepwise Activation of the Pro-Apoptotic Protein Bid at Mitochondrial Membranes. *Cell Death Differ* **2021**, *28* (6), 1910–1925.

(2) Hung, C.-L.; Lin, Y.-Y.; Chang, H.-H.; Chiang, Y.-W. Accessing Local Structural Disruption of Bid Protein during Thermal Denaturation by Absorption-Mode ESR Spectroscopy. *RSC Adv.* **2018**, *8* (60), 34656–34669.

(3) Morrison, K. L.; Weiss, G. A. Combinatorial Alanine-Scanning. *Current Opinion in Chemical Biology* **2001**, *5* (3), 302–307.

(4) Cunningham, B. C.; Wells, J. A. High-Resolution Epitope Mapping of hGH-Receptor Interactions by Alanine-Scanning Mutagenesis. *Science* **1989**, *244* (4908), 1081–1085.

(5) Tang, Q.; Fenton, A. W. Whole-protein Alanine-scanning Mutagenesis of Allostery: A Large Percentage of a Protein Can Contribute to Mechanism. *Human Mutation* **2017**, *38* (9), 1132–1143.

(6) Dengler, M. A.; Robin, A. Y.; Gibson, L.; Li, M. X.; Sandow, J. J.; Iyer, S.; Webb, A. I.; Westphal, D.; Dewson, G.; Adams, J. M. BAX Activation: Mutations Near Its Proposed Non-Canonical BH3 Binding Site Reveal Allosteric Changes Controlling Mitochondrial Association. *Cell Reports* **2019**, *27* (2), 359-373.e6.

(7) Schneider, C. A.; Rasband, W. S.; Eliceiri, K. W. NIH Image to ImageJ: 25 Years of Image Analysis. *Nat Methods* **2012**, *9* (7), 671–675.

(8) Humphrey, W.; Dalke, A.; Schulten, K. VMD: Visual Molecular Dynamics. *Journal of Molecular Graphics* **1996**, *14* (1), 33–38.

(9) Huang, J.; Rauscher, S.; Nawrocki, G.; Ran, T.; Feig, M.; De Groot, B. L.; Grubmüller, H.; MacKerell, A. D. CHARMM36m: An Improved Force Field for Folded and Intrinsically Disordered Proteins. *Nat Methods* **2017**, *14* (1), 71–73.

(10) Adcock, S. A.; McCammon, J. A. Molecular Dynamics: Survey of Methods for Simulating the Activity of Proteins. *Chem. Rev.* **2006**, *106* (5), 1589–1615.

(11) Baron, R.; Hünenberger, P. H.; McCammon, J. A. Absolute Single-Molecule Entropies from Quasi-Harmonic Analysis of Microsecond Molecular Dynamics: Correction Terms and Convergence Properties. *J. Chem. Theory Comput.* **2009**, *5* (12), 3150–3160.

(12) Polyansky, A. A.; Kuzmanic, A.; Hlevnjak, M.; Zagrovic, B. On the Contribution of Linear Correlations to Quasi-Harmonic Conformational Entropy in Proteins. *J. Chem. Theory Comput.* **2012**, *8* (10), 3820–3829.

(13) Šimėnas, M.; O'Sullivan, J.; Zollitsch, C. W.; Kennedy, O.; Seif-Eddine, M.; Ritsch, I.; Hülsmann, M.; Qi, M.; Godt, A.; Roessler, M. M.; Jeschke, G.; Morton, J. J. L. A Sensitivity Leap for X-Band EPR Using a Probehead with a Cryogenic Preamplifier. *Journal of Magnetic Resonance* **2021**, *322*, 106876.

(14) Keeley, J.; Choudhury, T.; Galazzo, L.; Bordignon, E.; Feintuch, A.; Goldfarb, D.; Russell, H.; Taylor, M. J.; Lovett, J. E.; Eggeling, A.; Fábregas Ibáñez, L.; Keller, K.; Yulikov, M.; Jeschke, G.; Kuprov, I. Neural Networks in Pulsed Dipolar Spectroscopy: A Practical Guide. *Journal of Magnetic Resonance* **2022**, *338*, 107186.

(15) Abramson, J.; Adler, J.; Dunger, J.; Evans, R.; Green, T.; Pritzel, A.; Ronneberger, O.; Willmore, L.; Ballard, A. J.; Bambrick, J.; Bodenstein, S. W.; Evans, D. A.; Hung, C.-C.; O'Neill, M.; Reiman, D.; Tunyasuvunakool, K.; Wu, Z.; Žemgulytė, A.; Arvaniti, E.; Beattie, C.; Bertolli, O.; Bridgland, A.; Cherepanov, A.; Congreve, M.; Cowen-Rivers, A. I.; Cowie, A.; Figurnov, M.; Fuchs, F. B.; Gladman, H.; Jain, R.; Khan, Y. A.; Low, C. M. R.;

Perlin, K.; Potapenko, A.; Savy, P.; Singh, S.; Stecula, A.; Thillaisundaram, A.; Tong, C.; Yakneen, S.; Zhong, E. D.; Zielinski, M.; Žídek, A.; Bapst, V.; Kohli, P.; Jaderberg, M.; Hassabis, D.; Jumper, J. M. Accurate Structure Prediction of Biomolecular Interactions with AlphaFold 3. *Nature* **2024**, *630* (8016), 493–500.
(16) Medema, J. P. FLICE Is Activated by Association with the CD95 Death-Inducing Signaling Complex (DISC). *The EMBO Journal* **1997**, *16* (10), 2794–2804.
(17) Watt, W.; Koeplinger, K. A.; Mildner, A. M.; Heinrikson, R. L.; Tomasselli, A. G.; Watenpaugh, K. D. The Atomic-Resolution Structure of Human Caspase-8, a Key Activator of Apoptosis. *Structure* **1999**, *7* (9), 1135–1143.
(18) Tessmer, M. H.; Stoll, S. chiLife: An Open-Source Python Package for in Silico Spin Labeling and Integrative Protein Modeling. *PLoS Comput Biol* **2023**, *19* (3), e1010834.
